# Long‐Term Demography of Spotted Hyena ( *Crocuta crocuta* ) in a Lion‐Depleted but Prey‐Rich Ecosystem

**DOI:** 10.1002/ece3.71025

**Published:** 2025-03-27

**Authors:** S. Martens, S. Creel, M. S. Becker, G. Spong, D. Smit, E. Dröge, J. M'soka, B. Mayani‐Nkhoma, T. Mukula, S. Mwaba, H. Ndakala

**Affiliations:** ^1^ Zambian Carnivore Programme Mfuwe Zambia; ^2^ Molecular Ecology Group SLU Umeå Sweden; ^3^ Department of Ecology Montana State University Bozeman Montana USA; ^4^ LUKE Helsinki Finland; ^5^ Wildlife Conservation Unit Oxford University Oxford UK; ^6^ U.S. Agency for International Development Lusaka Zambia; ^7^ African Parks Zambia Liuwa Plain National Park Kalabo Zambia; ^8^ Worldwide Fund for Nature Lusaka Zambia; ^9^ Zambian Department of National Parks and Wildlife Chilanga Zambia

**Keywords:** competitive release, ecosystem recovery, hyena survival, interspecific competition, large carnivore guild, population density, population dynamics, spotted hyena

## Abstract

Interspecific competition has strongly shaped the evolution of large carnivore guilds. In Africa, the lion ( *Panthera leo* ) and spotted hyena (*Crocuta crocuta, hereafter hyena*) exert direct and indirect competitive impacts on each other and on subordinate guild members. The impacts of competition on demography are complex and not well understood. With carnivore guilds now ubiquitously impacted by humans, disentangling the effects of interspecific competition and other drivers of hyena demography is important. Western Zambia's Greater Liuwa Ecosystem (GLE) provides a unique natural experiment where lions were functionally eliminated from the system. Hyenas are the apex predator, with an abundant prey base and low levels of human–hyena conflict. We measured GLE hyena survival and density using mark–recapture models fit to 10 years of data from 663 known individuals in 11 clans. GLE hyena densities were high, though slightly lower than other wildebeest‐dominated systems, and stable over 10 years. Survival rates were high for all age‐sex classes, and higher than those of other systems with high lion density, suggesting the possibility of competitive release from lion competition. These findings provide insights into long‐term hyena demography in the absence of their top competitor but with an abundant prey base. As humans continue to alter ecosystems and fundamental ecological relationships such as interspecific competition, altered dynamics such as competitive release are likely to be widespread and should be a focus of future research.

## Graphical abstract

Demography study using Bayesian methods to provide insights into long‐term spotted hyena ( *Crocuta crocuta* ) demography, in the absence of their top competitor, the lion ( *Panthera leo* ) but with an abundant prey base. This study illustrates the important manner in which humans are altering ecosystems by altering fundamental ecological relationships such as interspecific competition. As human pressure on large carnivore guilds continues to increase globally, recovering systems, such as the GLE, provide key insights into the subtle, but important ways ecosystems change when carnivore guild dynamics are altered.

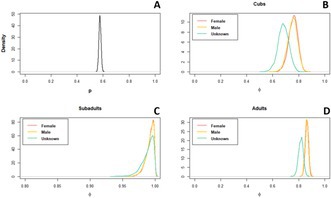

## 1 Introduction

**FIGURE 1:**
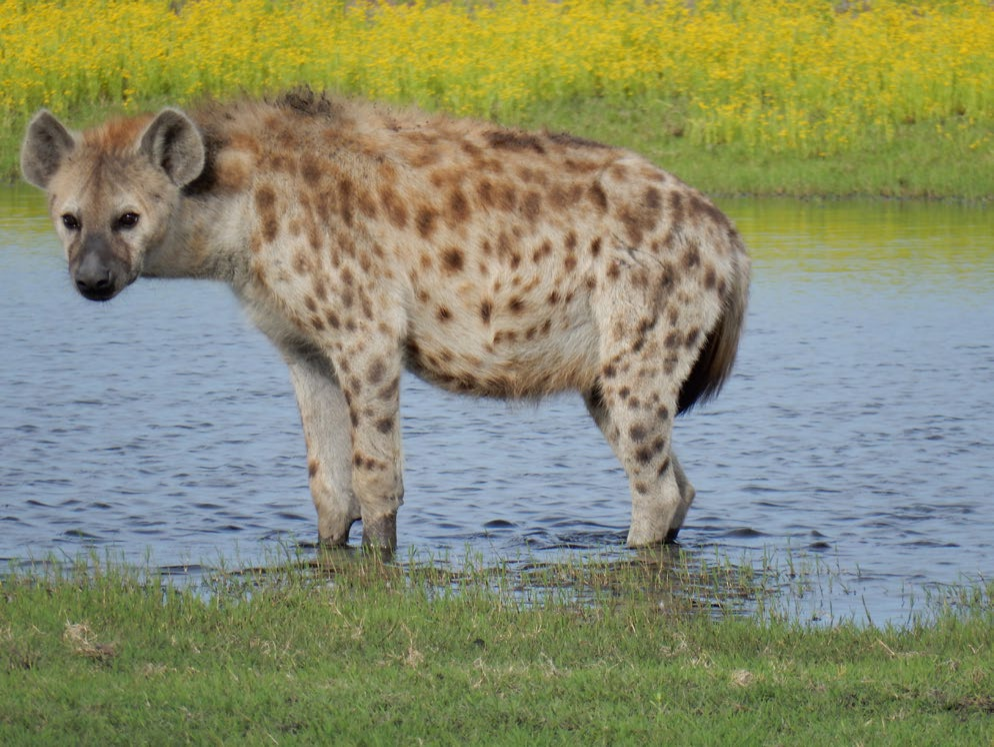
Adult female spotted hyena ( *Crocuta crocuta* ) in a seasonal pan. Survival across age and sex classes was high in this system with few lions and an abundant preybase.

Large carnivores are declining globally due to an array of human impacts, including habitat loss, prey depletion, and conflict (Ripple et al. 2014; Davis et al. 2022). In Africa, the lion ( *Panthera leo* ), spotted hyena ( *Crocuta crocuta* ; hereafter hyena; Figure 1), African wild dog ( *Lycaon pictus* ), cheetah ( *Acinonyx jubatus* ), and leopard ( *Panthera pardus* ) form the large carnivore guild. Within it, strong interference competition and intraguild predation, with the dominant competitors lion and hyena (Périquet et al. 2015; Creel et al. 2019), exert direct and indirect impacts on both species and their subordinate competitors (Creel and Creel 1996; Durant 2000; Dröge et al. 2017b). Lions and hyenas overlap significantly in range, habitat, diet, activity patterns, and space use, resulting in intense competition between the two species (Creel and Creel 1996; Hayward 2006; Périquet et al. 2015). As the human impacts of the Anthropocene continue to alter ecosystems worldwide, understanding and addressing alterations to carnivore guild interactions is an important area of research (Becker et al. 2024; Creel et al. 2023).

The spotted hyena is the most widespread and abundant large African carnivore (Watts and Holekamp 2008). Hyenas live in “clans” fission‐fusion, strongly hierarchical social systems composed of matrilineal family groups (Kruuk 1972; Holekamp and Dloniak 2010). Females usually stay within the same (natal) clan for life, while males usually disperse to another clan prior to breeding (Frank 1986; Höner et al. 2005; Holekamp and Dloniak 2010; Holekamp et al. 2012).

While the other guild species are IUCN‐listed as Endangered or Vulnerable, hyenas are listed as Least Concern (Böhm and Höner 2015). However, their distribution is fragmented, the continental population is likely declining, and rigorous demographic data are lacking in most ecosystems (Böhm and Höner 2015). Increasing evidence shows that hyenas are significantly impacted by the same anthropogenic threats as their sympatric competitors and may be more susceptible to some threats, such as snaring and conflict (Holekamp and Dloniak 2010; Hoffmann and Montgomery 2022; Benhaiem et al. 2022; Becker et al. 2024). In addition, more than their sympatric competitors, hyenas are vilified by humans, limiting and biasing support for conservation compared to other guild species (Hoffmann and Montgomery 2022) and persecuted across their range due to perceived associations with witchcraft (Dunham 2006; Somerville 2021). While little research exists, trophy hunting of hyenas likely causes significant risk of harvesting adult females given difficulties in sexing individuals, particularly when access to baits may be influenced by dominance and rank. These impacts are compounded by hyenas' large and protracted investment in maternal care and low fecundity (Watts and Holekamp 2009), limiting scope for demographic compensation of anthropogenic mortality. Knowledge of hyena demography and population dynamics largely comes from seminal studies in the East‐African Serengeti–Mara ecosystem (Wilkinson et al. 2024; e.g. Holekamp and Dloniak 2010; Watts and Holekamp 2008, 2009; Hofer and East 2003, 2008; Höner et al. 2005; Kruuk 1972). However, these studies cover small sections of the hyena range and habitat, making range‐wide inferences, particularly across human‐impacted landscapes, difficult.

Here we utilize a rare natural experiment in Western Zambia's Greater Liuwa Ecosystem (GLE) to quantify long‐term hyena demography under competitive release from lions in an ecosystem with abundant prey, relatively homogenous habitat, and low levels of human‐hyena conflict (M'soka et al. 2016). We estimated age‐sex specific hyena survival, density, and population trends from 2010 to 2019 in a tractable long‐term study of GLE hyenas, their competitors, and prey. Understanding the impacts of competitive release from lions on hyena demography provides key conservation insights into drivers of hyena dynamics, particularly as human impacts increase across virtually every remaining ecosystem for both species.

## 2 Methods

### 2.1 Study Site

Western Zambia's Greater Liuwa Ecosystem (GLE; S14.65583, E22.63828) comprises Liuwa Plain National Park (LPNP) and the surrounding Upper‐West Zambezi Game Management Area (GMA). LPNP, spanning 3660 km^2^, consists of seasonally flooded grassland interspersed with seasonal water pans, some permanent water sources, and open broadleaved woodland patches (M'soka et al. 2016; Creel et al. 2017). The GLE has a distinct wet season (December–April) characterized by extensive rainfall (approx. 1100 mm/year) and flooding, and a dry season (May–November) (M'soka et al. 2016). During the study, about 16,500 registered human residents lived in the GLE (African Parks Network 2018).

The GLE supports the second largest blue wildebeest ( *Connochaetes taurinus taurinus* ) population in Africa (African Parks Network 2018; Watson et al. 2022). Migrations of the most abundant herbivore species (see below) cause seasonal differences in prey availability between the Southern‐central and Northern parts of the GLE. The former is a high hyena density area, whereas the northern parts of the GLE support lower hyena densities (Watson et al. 2022). Wildebeest are the primary prey for GLE hyena (Dröge et al. 2017b), comprising 92% of kills, and hyena predation is the primary limiting factor for wildebeest survival (Watson et al. 2022). Wildebeest densities range from 6.2 to 60.8 individuals/km^2^ (M'soka et al. 2017) and the area supports populations of zebra ( *Equus quagga* ; 1.8–8.1 individuals/km^2^), oribi ( *Ourebia ourebi* ; 1.1–14.5 individuals/km^2^), and other herbivore species in smaller numbers (M'soka et al. 2017; African Parks Network 2018).

The GLE's carnivore guild structure is unusual after decades of depletion through poaching and conflict. Leopards are absent, and lions were nearly absent from the system, save for one lioness, for several years prior to this study. After subsequent reintroductions of two, two, and one lion in 2009, 2011, and 2016 respectively, the population increased to 10 individuals by 2019. The near absence of lions (< 0.3 individuals/100 km^2^, more than an order of magnitude less than other wildebeest‐dominated ecosystems; Creel, unpublished data; Durant et al. 2011; Elliot and Gopalaswamy 2017) in a system with a large prey base has largely released the hyena population from lion competition and predation, allowing the hyena to become the apex predator. A small but stable population of cheetah (15–20 known individuals) is present. African wild dogs, primarily in one pack, were present until 2014 when they were locally extirpated, likely due to rabies (M'soka et al. 2016), and remained absent during this study. The apex predator population, its competitors, and all major prey species are intensively studied and their dynamics well described, allowing for comprehensive analysis of hyena demography and dynamics (See Christianson et al. 2018; Creel 2018; Creel et al. 2017, 2019; Dröge et al. 2017a, 2017b, 2019; M'soka et al. 2016, 2017).

### 2.2 Data Collection

We used a combination of stratified random sampling and opportunistic sightings to observe individually known hyenas (*n* = 663) within the study area. We aimed to observe each collared individual (*n* = 1–3 adult females per clan depending on the size of the clan) every 2 weeks and recorded all sightings of hyenas and other large carnivores. Given that hyenas are highly social, focal monitoring of collared individuals enabled effective monitoring of uncollared clan members throughout the year. Observations were conducted from vehicles and motorbikes and started upon sighting of an individual or group. Observations lasted from 1 min to 12 h depending on the behavior of the individual or group and focal data collection. Accessible clan communal dens were visited regularly (every 2 to 10 days) to monitor cub recruitment and survival. Observations at dens and searches for collared individuals were done in the morning (06.00–11.00 h) and evening (16.00–20.00 h); opportunistic sightings were recorded at any time. Night‐time observations happened between 17.00 and 09.00 h, in which we followed a focal collared female and recorded hunting and social behavior and kills. Kills often attracted large groups of hyenas (up to 47 individuals), often allowing identification of individuals. Observations in the wet season, when the plains were flooded and less accessible, were generally restricted to the central portion of the LPNP and focused on six clans in the core area of the park. The dry season allowed for the observation of 11 clans across the GLE, overlapping with the range of the migrating wildebeest population (M'soka et al. 2016; Creel et al. 2017).

### 2.3 Field Methods

Each hyena has a unique spot pattern that can be used to identify individuals. As per M'soka et al. (2016) we photographed and identified each individual encountered. We collected > 45,000 identification photos. For each sighting, we identified individuals by comparing photos to our database of identification photos and finding a matching spot pattern. Young cubs were identified through association with the mother. Individuals were assigned to clans based on communal den visitation, individuals with which they were observed cooperating in hunting, and spatial association with other known clan members (Kruuk 1972).

Most individuals were frequently re‐sighted, and sex was assigned by the shape of the end of the phallic glans (Frank et al. 1990), body outline, size, reproductive status, visible mammary glands, or a combination thereof (Frank 1986). We included an unknown sex class in our analysis for individuals whose sex could not be determined using these criteria.

We assigned a date of birth to each individual born during the study based on the color and development of the coat, body size, facial features, and behavior of juveniles. Individuals first identified at older ages were assigned a date of birth based on body size, shape and size of head, scars, and coat texture. We assigned an error range relative to the current age class of an individual to each assigned date of birth (M'soka et al. 2016). Because of uncertainty in estimated ages, particularly for adults, our analysis considered three age classes that can be determined with little uncertainty.

One to three adult females were fitted with a VHF collar within each clan continuously (with brief exceptions) throughout the study period. Collared females (*n* = 44) enabled regular detections of other clan members through association at dens, kills, resting sites, and hunts. When a new clan was included in the study, we collared at least one female as soon as possible. We aimed to re‐collar the same individuals (typically within 3 years of collar activation) to maximize data on collared individuals' life histories. Sixty‐two VHF and 10 GPS radio collars (Telonics, Advanced Telemetry Solutions and Africa Wildlife Tracking) were deployed over the course of the study. From 2010 to 2013, individuals were darted and radio‐collared by experienced personnel, authorized by the Department of Veterinary and Livestock Development and the Zambian Department of National Parks and Wildlife. From 2013 onwards, darting and collaring were done by Zambian registered veterinarians. Protocols were also approved by the MSU IACUC. Individuals were typically darted using a Daninject JM CO_2_ rifle with a combination of medetomidine and tiletamine‐zolazepam (tz), reversing the medetomidine with antipamezole when the tz began to wear off. Individuals were monitored until the animal was fully awake, alert, and moving normally.

### 2.4 Data Analysis

#### 2.4.1 Annual Survival Rates

We compiled individual detection histories from June 2010–November 2019 for 663 hyenas, yielding 22,394 detections. We binned detections into 2‐month occasions to obtain a 57‐occasion encounter history for each individual. We used Bayesian methods to fit Cormack‐Jolly‐Seber (CJS) models of age‐class‐ and sex‐specific annual apparent survival rates (*φ*) allowing for individual heterogeneity in the probability of detection (*p*) (Seber 1965; Royle 2008; Pledger et al. 2010; Kéry and Schaub 2012). Age classes were defined as cub (0–1 years old), subadult (1–3 years), and adult (> 3 years; Frank 1986; Tanner et al. 2010). As in prior studies (Rosenblatt et al. 2014; Goodheart et al. 2021; Creel et al. 2024) we tested for an effect of collaring on survival for hyenas and confirmed that the mortality rate of radio‐collared individuals was not higher than that of uncollared individuals.

CJS models estimate apparent survival probabilities (*φ*) after accounting for the probability of detection (*p*), allowing for an overlapping set of variables to affect these parameters (Pledger et al. 2003; Royle 2008; Kéry and Schaub 2012; Goodheart et al. 2021). The model provides an estimate of survival conditional on first detection, allowing for individuals to enter the study in a staggered way. Individuals that were known to have died during the study were excluded from the analysis as they were not a substantial portion of the total sample (*n* = 13) and their inclusion would have required a combination of recovery and recapture models (Burnham 1993; Rosenblatt et al. 2014). We used the R2jags (Yu and Yajima 2012) package in R to construct a model with fixed effects of sex and age on survival rates, allowing for variation between individuals in the detection probability through individual random effects with a Gaussian distribution on the logit scale (Kéry 2010). Using uninformative uniform prior distributions for both parameters, with bounds as recommended by Kéry and Schaub (2012), we fit the model with three Markov chains, running 5000 iterations after a burn‐in of 500 steps. We confirmed good model fit by examining trace plots and confirming that R‐hat was close to one, and effective sample size was close to nominal for all parameters, by confirming that a logit‐normal function provided a good fit to the observed distribution of detection probabilities, and by inspecting Q‐Q plots.

Cubs were first detected at the average age of 2.41 months (≈0.2 years), so we annualized the 2‐month survival rate of cubs by exponentiation to the power of (6 * (12/12–9.59)). To further check the reliability of the model‐generated cub survival rates, we compared them to a manually calculated approximate cub mortality rate. For this verification calculation, we assumed that *p* = 1 for den‐dwelling cubs (all individuals in the cub age‐class), if an individual disappeared while in the cub age‐class, it was considered a known death. We divided the number of individuals first detected in the cub age‐class and sighted at least once in a subsequent age‐class by the total number of individuals first detected in the cub age‐class to get an approximate annual cub mortality rate.

#### 2.4.2 Estimating Population Density

We fit closed mark‐recapture models using Bayesian methods to fit closed capture models of hyena abundance in the wet season (December–April) and dry season (May–November) in each year (Royle et al. 2007; Kéry and Schaub 2012). We fit and compared two abundance models, one that modeled individual heterogeneity effects on the detection probability, *p* (Huggins 1989), and one without individual heterogeneity. Both models were fit using Royle et al.'s data augmentation approach (Royle et al. 2007). Using DIC scores to compare, we found that these supported the model with individual heterogeneity in *p* (again modeled with an individual random effect that was logit‐normal) (∆DIC scores > 10 for all years and seasons). To account for seasonal and annual variation, we aggregated each season's detection histories into 1‐month bins. We created capture histories with five (wet season) and seven (dry season) occasion encounter histories for each individual per year, yielding abundance estimates for 10 dry seasons (2010–2019) and 9 wet seasons (2011–2019). We converted abundance to a density estimate ($\hat{D}$) by dividing the seasonal estimate of population size ($\hat{N}$) by the study area size in that season ($\hat{A}$). Seasonal study area size was estimated as the 90th percentile isopleth of a kernel utilization distribution (KUD; Worton 1989) fit to all sighting locations (GPS coordinates) of all individuals included in the density estimate for that season, using the R package adehabitatHR (Calenge 2022).

## 3 Results

We identified 663 individual hyenas from 11 clans and one group of individuals not assigned to a clan at the end of the study, across the GLE. We observed 114–252 (mean = 174) hyenas per season. Table 1 contains an overview of the number of animals in each sex class and age class at first sighting. Of the 13 hyenas that were known to have died, four were observed to be killed by lions. Other deaths may have been caused by starvation or infanticide/siblicide (all juveniles), but the cause of death could not be established with full certainty.

**TABLE 1:** Number of animals per sex‐class that were first sighted in each age‐class.

	Cub	Sub‐Adult	Adult	Total
Female	81	21	83	185
Male	83	24	86	193
Unknown	145	42	85	272
Total	309	87	254	650

### 3.1 Population Density, and Growth Rate

**FIGURE 2:**
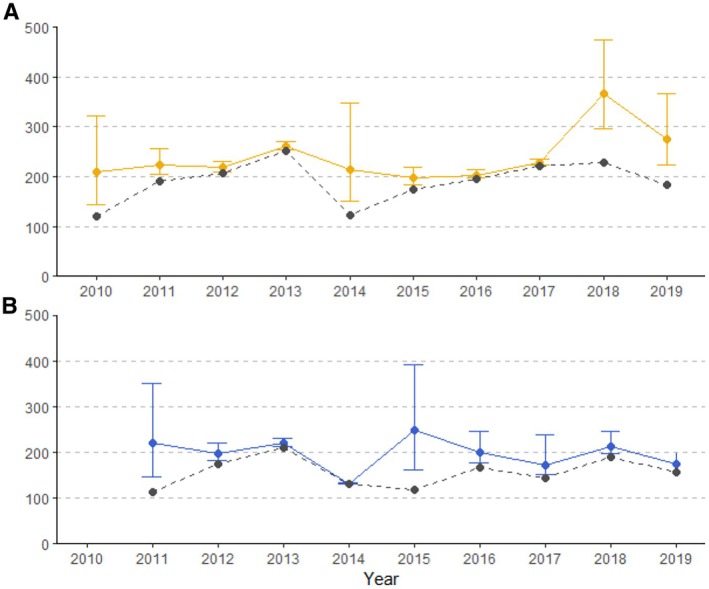
Observed study population numbers (dashed) and population estimates (solid) from the closed capture model per year for dry season (A) and wet season (B). Error bars show 95% credible intervals.

Population density was relatively high and stable with, overall, no clear pattern of increase or decrease. In the first season of the study (dry season 2010) we observed 120 hyenas and estimated a population size of 208 individuals (95% CRI: 145–322) in a study area size of 240.2 km^2^, yielding an estimated density of 0.87 hyenas/km^2^. In the last season of the study (dry season 2019) we observed 183 hyenas and estimated a population size of 276 (95% CRI: 223–366) individuals in a study area of 1508.8 km^2^, giving the lowest density estimate of 0.18 hyenas/km^2^. Population estimates per season and observed population counts (with no correction for probability of detection) from the closed capture model are shown in Figure 2.

**FIGURE 3:**
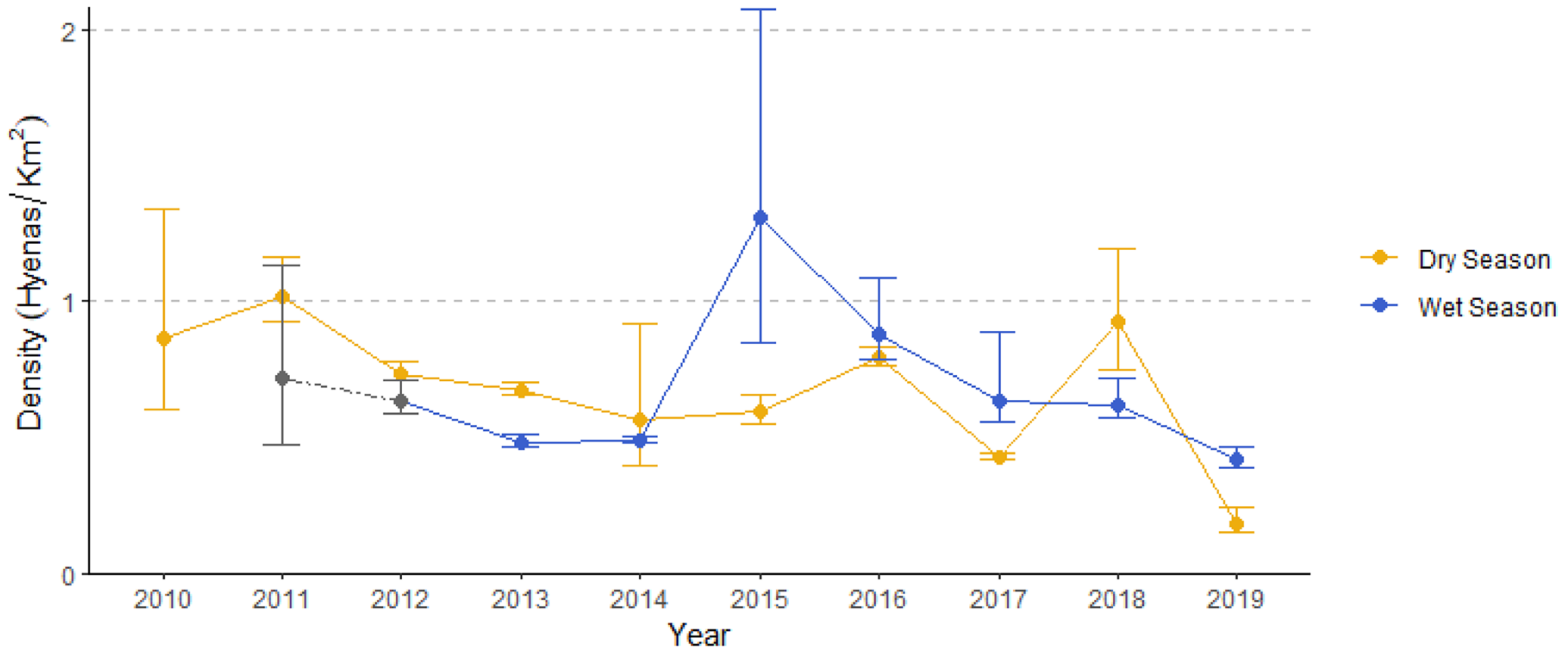
Density estimates ($\hat{D}$) per season and per year for 2010–2019 including 95% credible intervals, following from the division of the through CJS models estimated population size ($\hat{N}$) by the 90th percentile isopleth KUD estimate of study area size ($\hat{A}$). The gray points and dashed gray line for wet season years 2011 and 2012 are estimated using mean study area size of the 2013–2019 wet seasons.

Density estimates in dry seasons from 2010 to 2019 ranged from 0.18 to 1.01 hyenas/km^2^ (Figure 3); mean dry season density was 0.68 (95% CRI: 0.59–0.83) hyenas/km^2^. For wet season estimates in 2011 and 2012, we had no good basis for estimating the study area based on hyena sighting locations. We therefore divided the 2011 and 2012 wet season population estimates by the mean wet season study area size for 2013–2019, which we considered the most representative estimate of the area occupied by the observed hyenas in those years.

**FIGURE 4:**
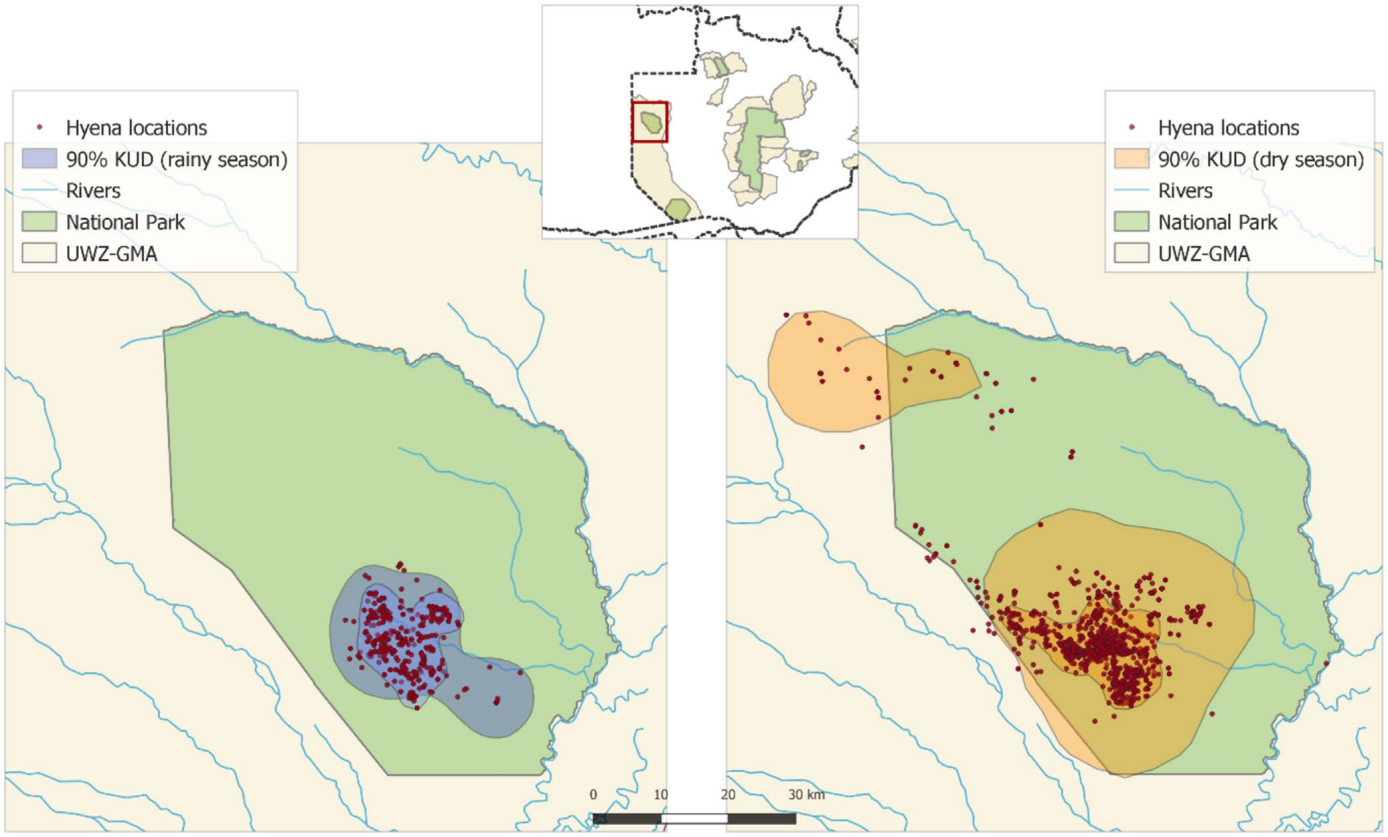
Smallest and largest study area estimates following from the 90th percentile isopleth KUD for the rain season (2015 and 2013; left) and the dry season (2011 and 2019; right).

Estimates of density for the wet season (2011–2019) ranged between 0.42 and 1.31 hyenas/km^2^ (Figure 3), mean = 0.69 (95% CRI: 0.57–0.90) hyenas/km^2^. Wet season 2015 had the highest density estimate, but also the smallest study area size estimate (190 km^2^). The differences in study area sizes are illustrated in Figure 4. Although mean density is similar for dry and wet seasons, the average population estimate was usually higher for the dry season than the wet season, due to a combination of larger population estimates and smaller study areas in the wet season.

### 3.2 Age‐ and Sex‐Specific Annual Survival

**FIGURE 5:**
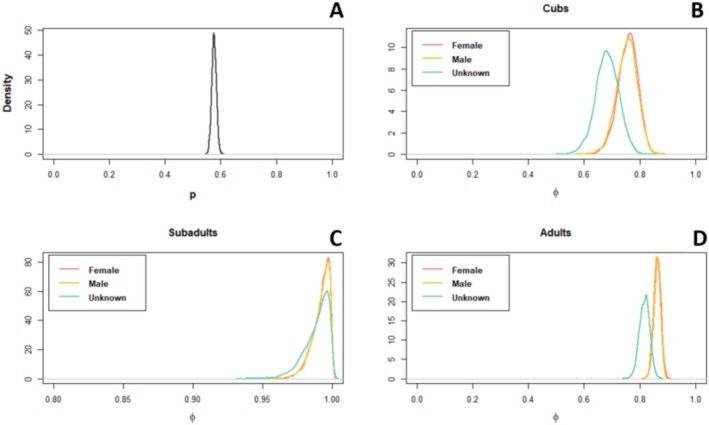
(A) Posterior probability distributions for the mean probability of detection per 2‐month time bin. Posterior probability distributions for annual survival rates (*φ*) of hyena cubs (B), sub‐adults (C), and adults (D) as estimated by a Cormack–Jolly–Seber model fit to data spanning 2010–2010.

Annual survival rates across all sex and age classes were high. Estimated annual survival rates for sub‐adults were highest at 0.99 (95% CRI: 0.97–1.00) for males and females. Across all age and sex classes, cub survival was lowest with *ɸ* = 0.76 (95% CRI: 0.68–0.82) for females, 0.75 (95% CRI: 0.68–0.78) for males, and 0.69 (95% CRI: 0.60–0.71) for individuals of unknown sex. Mean adult survival for females and males was 0.86, with narrow credible intervals (95% CRI: 0.84–0.89) and 0.82 (95% CRI: 0.78–0.83) for unknown sex individuals (Figure 5). Mean bi‐monthly probability of detection (*p*) was 0.575 (Figure 5). A model with only sex effects on detection gave *p* for females and males as 0.640 and 0.601, respectively, but *p* was 0.497 for the unknown sex class, reflecting that individuals who were detected more often were more likely to be assigned a sex.

In total, 309 individuals entered the study at age 0–0.99 years (the cub age‐class). Of these cubs, 279 could have reached the age of 1 year (entered sub‐adult age‐class) before the end of the study. Of this group, 53 individuals were never seen after the time they would technically have reached the age of 1 year. This yields an estimated mortality of 23.5% for cubs, comparable to the CJS model estimates.

## 4 Discussion

Spotted hyenas are a widespread species with strong ecological impacts but are increasingly impacted by humans. Despite these factors, rigorous studies of hyena demography and the ecological and anthropogenic factors affecting it are rare, and consequently, these dynamics are not well understood. Our study provided rigorous, long‐term demographic data for a key stronghold population while evaluating hyena dynamics in the absence of their top competitor but with the presence of abundant prey. As expected in conditions of low competition and high prey abundance, we found a high population density in our study population, though with no clear trend for increase or decrease and high annual survival rates for all age and sex classes. While we cannot be certain as to the drivers of these observed dynamics, this study provides a baseline with which to further evaluate changes in lion, prey, and hyena populations as they occur. Collectively, this provides significant insights into hyena demography and the impacts of humans on carnivore guild dynamics.

### 4.1 Population Density

Hyena density in the GLE was relatively high (dry season mean = 0.68, CRI = 0.59–0.83; wet season mean = 0.69, CRI = 0.57–0.90) when compared to other ecosystems (see below), and appeared to be relatively stable without significant increases or decreases over the study period. M'soka et al. (2016) reported densities of 0.13–0.52 hyenas/km^2^ over 2010–2013, for a central portion of the area that we examined, which included the ranges of five clans. Our 2010–2019 estimates and overall means are consistently higher, emphasizing the value of long‐term monitoring efforts for large carnivore research, and supporting Rosenblatt et al.'s (2014) assessment that accuracy of estimates from intensive monitoring improves with time. Mean densities were slightly lower than estimates from other wildebeest‐dominated savannah ecosystems such as Masai Mara NR, Kenya (0.95 hyenas/km^2^; Watts and Holekamp 2008) and Ngorongoro Crater CA, Tanzania (mean adult hyena density: 0.89 ± 0.26; Höner et al. 2005). Our estimates are higher than other, non‐wildebeest‐dominated, systems, like central Tuli, Botswana (short‐term camera trap survey; 0.149 ± 0.022 hyenas/km^2^; Vissia et al. 2021) and Ongava GR, Namibia (0.081 hyenas/km^2^; Stratford et al. 2019). Interestingly, high densities were reported for Amboseli NP, Kenya (1.65 hyena/km^2^) with lion densities (0.079–0.135 lion/km^2^) lower than the Serengeti‐Mara. Factors such as high prey densities and minimal anthropogenic activity could facilitate high densities here (Watts and Holekamp 2008).

These numbers suggest that, other than interspecific competition, hyena densities are also strongly correlated with prey density and anthropogenic activity. Similar to reports for Serengeti–Mara, hyenas in the GLE maintain a high population density and high annual survival rates for all age‐sex classes (see below).

### 4.2 Survival Rates

Annual survival rates for GLE hyenas were high across all sex‐age classes, particularly notable for long‐lived mammals that typically have high adult survival and variable juvenile survival. Adult and sub‐adult survival were comparable to Ngorongoro Crater, where Höner et al. (2005) reported sub‐adult and adult mortality rates of 0.099 ± 0.043. Interestingly, estimated cub and sub‐adult survival to the age of 2 years was 35.6% in the Serengeti, which is lower than our GLE report, even though we categorized the sub‐adult age class as 1 year longer (Hofer and East 2003). A similar survival rate was reported for Masai Mara, with a mortality of 63% in the first 2 years (Watts and Holekamp 2009). Lion densities in the Serengeti (0.099–0.189 lions/km^2^ in 2005; Durant et al. 2011) and Masai Mara (0.17 lions/km^2^; Elliot and Gopalaswamy 2017) are higher than those of the GLE. As multiple studies report direct killing by lions as a leading source of mortality for hyenas of all age classes (Höner et al. 2005; Watts and Holekamp 2009; Périquet et al. 2015), higher GLE hyena survival is not surprising. Lower cub and sub‐adult survival rates in these areas are therefore likely related to greater interspecific competition (Périquet et al. 2015). Ngorongoro also supports higher lion densities, but the impact on hyena survival may be offset by the year‐round high prey densities (Höner et al. 2005).

While we observed lions killing hyenas infrequently during our study (*n* = 4), our survival estimates allow us to infer that the impact of lions was limited, as expected from their very low density. Furthermore, the success of the GLE population, despite limited scavenging opportunity, undermines the misconception that hyenas primarily scavenge, as the vast majority of food acquisition was through hunting (Dröge et al. 2017b; Creel et al. 2017; Watson et al. 2022). While scavenging provides an important food source for hyenas in most ecosystems (Périquet et al. 2015), the benefit of low competitor densities apparently exceeds the benefit of scavenging opportunities.

### 4.3 Study Limitations

There were several limitations to our study. We included unsexed individuals in our study due to the difficulties in sexing hyenas. When the individual remained of unknown sex, it was more likely that this individual had either died before sex could be assigned or was detected infrequently, providing less opportunity for sexing. As a consequence of lower survival rates for unsexed animals, estimated survival rates for males and females are likely biased slightly high. This issue is important mainly for cubs, who may have been seen few times or only in obscured conditions. However, censoring the unknown sex class from the analysis would not remove the potential bias; therefore, we retained unsexed individuals.

Time trends in population density are notoriously difficult to describe for large carnivores, and sampling errors in population estimates are hard to eliminate (Rosenblatt et al. 2014). Our density estimates pertain to a study area that expanded over the course of the study and that encompassed a migratory prey base in a seasonal environment. Consequently, utilizing the same study area estimate for each season would have disregarded important aspects of spotted hyena movement. We therefore estimated density for different areas in the wet and dry seasons to allow for seasonally fluctuating local prey densities that are typical for the GLE (M'soka et al. 2017; Watson et al. 2022). More clans were also added to the study over time, increasing our sample size over the study period. Consequently, at least a portion of the annual fluctuation in density estimates is likely due to this variability, though our estimates correct for probability of detection. For example, beginning in dry season 2019, we were able to consistently monitor in the Northern LPNP and GMA, increasing the study area. The lower density for this season probably reflects the uneven distribution and density of hyenas across the GLE.

### 4.4 Potential Drivers of GLE Hyena Demography

While most carnivore guild competition studies are conducted in relatively intact systems where the full species complement has persisted and human impacts are limited, this study provides the first long‐term detailed demography of Zambian spotted hyena populations. Initial studies of hyena demography in the GLE by M'soka et al. (2016) highlighted that the high survival and density of hyenas were likely attributable to the functional absence of a competing lion population, an abundant preybase, and limited conflict with humans. Our study reinforces those findings with additional data over a longer time period. Given their strong temporal, spatial, and dietary overlap, and intense interference competition through direct mortality and kleptoparasitism, lions are a significant limiting factor to hyena populations through competition (Creel and Creel 1996).

In the functional absence of lion competition, the high density and survival exhibited by hyenas could potentially be attributable to competitive release. However, prey abundance is also likely to play a key role in hyena demography and can potentially moderate competition and facilitate coexistence between the species (Périquet et al. 2015). The primary prey of GLE hyena, the blue wildebeest, remained relatively stable during the course of the study (Watson et al. 2022), while the lion population increased modestly from three to ten lions; thus, more longitudinal data in this system will be insightful.

A key question in GLE hyena demography is the trajectory of the population given the high survival and density. While we did not detect significant study population changes, it is possible there are population‐level changes in dispersal success and recolonization of formerly occupied range in the GLE and beyond that may account for this. Our study comprised the core GLE population, and density dependence may not yet be occurring at the density or population level as a result. Future studies will evaluate changes in clan composition, size, survival, and reproduction across the GLE concurrent with changes in competitor and prey populations.

### 4.5 Conclusion

As human pressure on large carnivore guilds continues to increase globally, recovering systems such as the GLE provide key insights into the subtle but important ways ecosystems change when carnivore guild dynamics are altered. Despite their role as apex predators, the ecological and anthropogenic drivers of hyena demography are poorly understood. Rangewide, populations remain understudied and in need of attention to understand the drivers of demography, evaluate threats, and address declines.

## Author Contributions

**S. Martens:** conceptualization (equal), data curation (lead), formal analysis (lead), investigation (equal), methodology (equal), visualization (lead), writing – original draft (lead), writing – review and editing (lead). **S. Creel:** conceptualization (lead), data curation (equal), formal analysis (supporting), funding acquisition (equal), methodology (lead), resources (equal), software (lead), supervision (lead), validation (equal), visualization (supporting), writing – original draft (supporting), writing – review and editing (supporting). **M. S. Becker:** conceptualization (lead), funding acquisition (lead), investigation (equal), methodology (equal), project administration (lead), resources (lead), writing – original draft (supporting), writing – review and editing (supporting). **G. Spong:** conceptualization (equal), supervision (equal), writing – original draft (supporting), writing – review and editing (supporting). **D. Smit:** data curation (supporting), investigation (equal). **E. Dröge:** investigation (supporting). **J. M'soka:** investigation (equal). **B. Mayani‐Nkhoma:** investigation (equal). **T. Mukula:** investigation (equal). **S. Mwaba:** investigation (equal). **H. Ndakala:** investigation (supporting).

## Conflicts of Interest

The authors declare no conflicts of interest.

## Supporting information


Table S1.

## Data Availability

Data and related Supporting Information are available on Dryad. DOI: 10.5061/dryad.612jm64d5, version 1. Reviewer link: https://datadryad.org/stash/share/Saau6fNz3QUbyh_5aCa2kk6bl138QBJeJwhe2SrEb4k.
