# Supplementary material for: Long‐Term Demography of Spotted Hyena ( Crocuta crocuta ) in a Lion‐Depleted but Prey‐Rich Ecosystem
Source: Ecol Evol. 2025 Mar 27;15(4):e71025. doi: 10.1002/ece3.71025 (PMC11949567; doi:10.1002/ece3.71025)
Supplement: Supplementary file 1 — Table S1. [file ECE3-15-e71025-s001.pdf]

Appendix I

Table i: Seasonal estimates of hyena population numbers and resulting densities when divided by the season's 90th percentile isopleth Kernel Utilisation Distribution to estimate study area. See methods for details.

	**Dry Season**			**Wet Season**		
Year	N.mean (95% CRI)	Area (km^2)	Density (hy/km^2)	N.mean (95% CRI)	Area (km^2)	Density (hy/km^2)
2010	208 (145-322)	240	0.87	-	-	-
2011	224 (204-257)	221	1.02	222 (147-351)	*311*	0.72
2012	218 (210-230)	296	0.74	197 (183-221)	*311*	0.63
2013	261 (254-271)	387	0.67	220 (213-232)	456	0.48
2014	215 (150-347)	379	0.57	131 (130-135)	268	0.49
2015	198 (183-219)	333	0.59	249 (161-394)	190	1.31
2016	203 (197-213)	256	0.79	200 (178-247)	227	0.88
2017	228 (223-235)	528	0.43	228 (223-235)	270	0.64
2018	367 (297-474)	398	0.92	367 (297-474)	342	0.62
2019	276 (223-366)	1509	0.18	276 (223-366)	420	0.42
**Mean**	**240 (209-293)**	**-**	**0.68**	**198 (169-251)**	**-**	**0.69**
